# Dr. Andrew Thomas Dunn

**Published:** 1907-06

**Authors:** 


### OBITUARY.

Dr. Andrew Thomas Dunn, of North Augusta, Ontario, died at his home January 16, 1907, after an illness lasting several years. He graduated at the Medical Department, University of Buffalo, in 1863, and at the Royal College of Physicians and Surgeons, Kingston, Ontario, in 1864.

